# Orthotopic heart transplantation with reconstruction of persistent left superior vena cava

**DOI:** 10.1186/s40792-020-00834-9

**Published:** 2020-04-15

**Authors:** Kazuma Handa, Hiroki Hata, Koichi Toda, Shigeru Miyagawa, Yasushi Yoshikawa, Daisuke Yoshioka, Yoshiki Sawa

**Affiliations:** Department of Cardiovascular Surgery, Osaka University Graduate School of Medicine, 2-2 Yamadaoka, Suita, Osaka, 565-0871 Japan

**Keywords:** Persistent left superior vena cava, Orthotopic heart transplantation

## Abstract

**Background:**

Persistent left superior vena cava is a not uncommon congenital vascular abnormality. We report a case of heart transplantation with reconstruction of persistent left superior vena cava using a prosthetic vascular graft.

**Case presentation:**

A 20-year-old man with idiopathic dilated cardiomyopathy and persistent left superior vena cava underwent orthotopic heart transplantation 2 years and 3 months after left ventricular assist device implantation. Because the persistent left superior vena cava had a larger diameter than the right superior vena cava, the transected persistent left superior vena cava was reconstructed with a prosthetic vascular graft anastomosed to the free wall of the right atrium. Postoperative enhanced computed tomography revealed good patency of the graft. The patient’s postoperative course has been uneventful during 2 years of follow-up, despite the risk of complications.

**Conclusions:**

Reconstruction of a persistent left superior vena cava with a prosthetic vascular graft may be one option at the time of heart transplantation.

## Background

Persistent left superior vena cava (PLSVC) is a not an uncommon congenital vascular abnormality (incidence of 0.3–0.5% in the general population and 3–10% among those with congenital heart anomalies) [1]. This condition is usually asymptomatic and does not cause hemodynamic changes [2]. Prior case reports have described orthotopic heart transplantation in patients with PLSVC. In some of these cases, simple ligation of the PLSVC was performed, whereas in others the PLSVC was reconstructed [3–5]. We herein report a case of PLSVC reconstruction using a prosthetic vascular graft in a patient undergoing heart transplantation.

## Case report

**Fig. 1:**
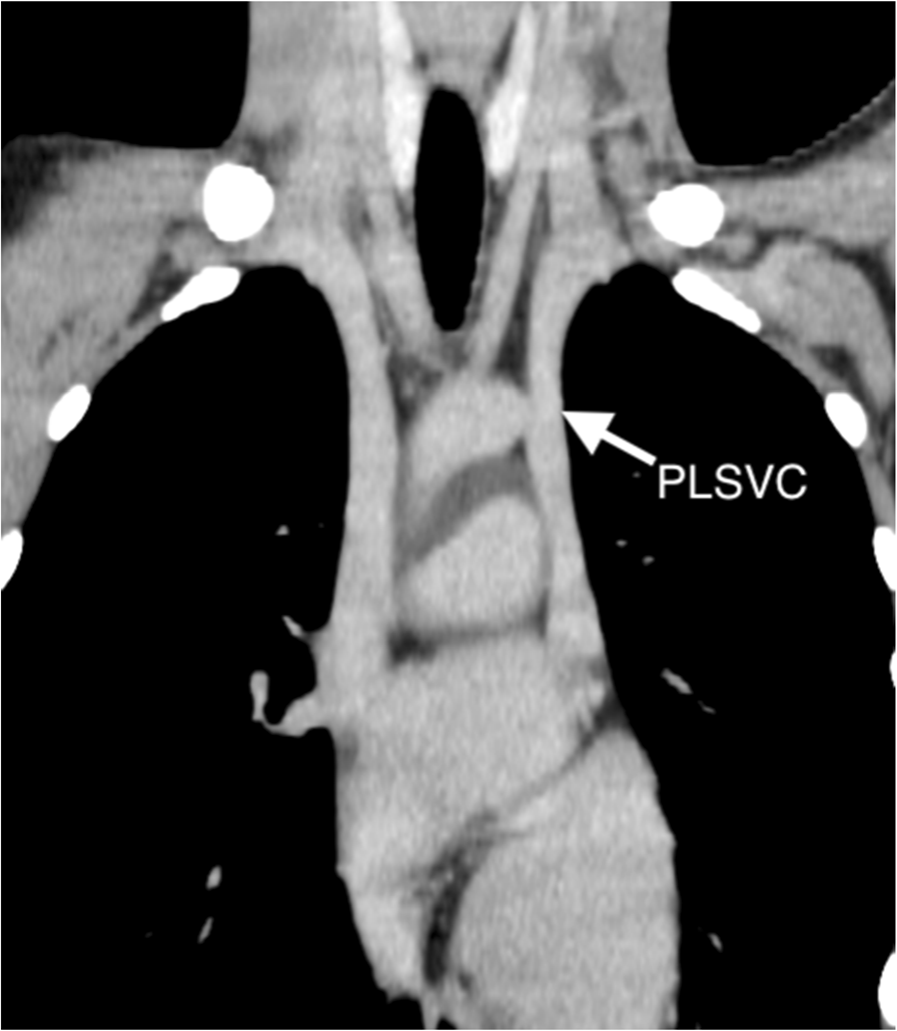
Preoperative computed tomography shows persistent left superior vena cava (PLSVC) and left brachiocephalic vein atresia

A 20-year-old man diagnosed with idiopathic dilated cardiomyopathy presented with deteriorated cardiac function (left ventricular (LV) ejection fraction 18%). After registration with the Japan Organ Transplant network, he underwent continuous-flow LV assist device (LVAD) implantation as a bridge to transplantation. When a donor in good condition was found 2 years and 3 months after LVAD implantation, we decided to perform heart transplantation. The PLSVC and atresia of the left brachiocephalic vein (LBCV) were recognized on preoperative chest computed tomography (Fig. 1). Because the right superior vena cava (SVC) seemed larger than the PLSVC, we initially planned to ligate the PLSVC.

After full median sternotomy and dissection around the heart, we found that, contrary to our expectations, the PLSVC had a larger diameter than the right SVC. Therefore, we decided to reconstruct the PLSVC instead of ligating it. We established cardiopulmonary bypass by inserting a 21-Fr cannula into the ascending aorta and 24-Fr and 28-Fr drainage cannulas into the right SVC and inferior vena cava, respectively. An additional 28-Fr cannula was inserted into the PLSVC.

**Fig. 2:**
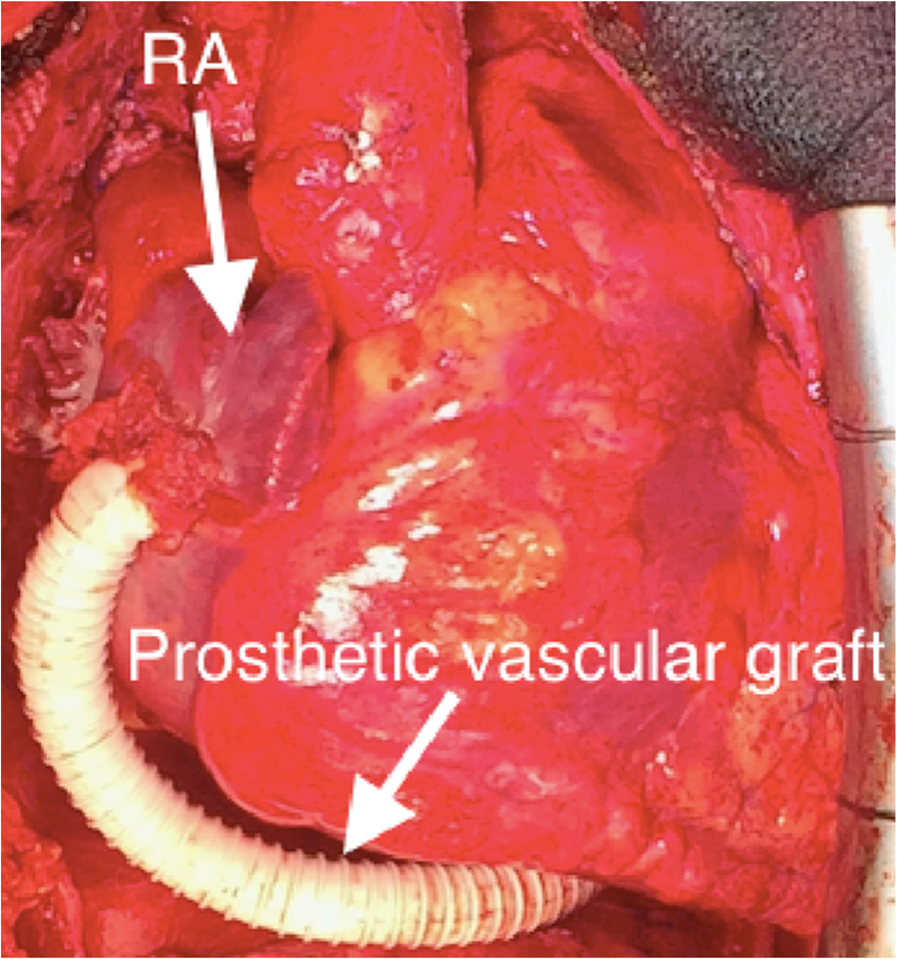
Intraoperatively, the prosthetic vascular graft from the PLSVC is anastomosed to the right atrium

**Fig. 3:**
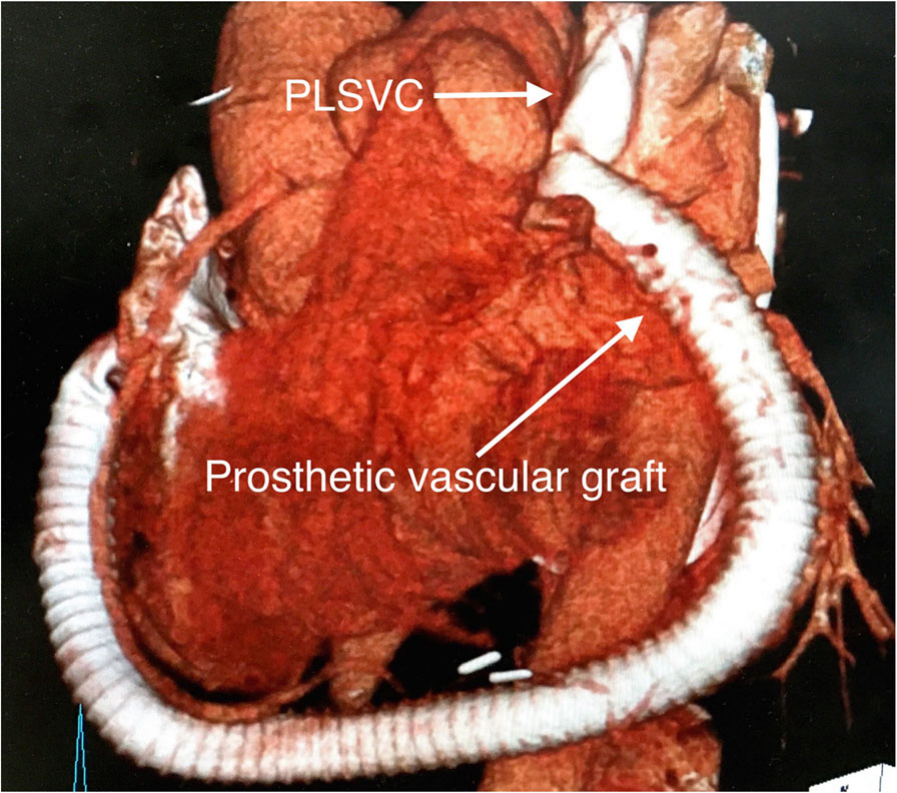
Postoperative CT angiography shows patency of the reconstructed PLSVC and prosthetic graft

Orthotopic heart transplantation was performed with modified bicaval technique. After completion of the anastomoses of the left atrium, pulmonary artery, ascending aorta, inferior vena cava, and right SVC, the transected PLSVC was reconstructed with a 10-mm ringed expanded polytetrafluoroethylene graft, which was passed behind the left ventricle and anastomosed to the free wall of the right atrium (RA) in side-to-end fashion (Fig. 2). The postoperative course was uneventful, and the patient did not experience any complications, including venostasis-related symptoms. He was discharged home 6 weeks after the transplantation. The prothrombin time international normalized ratio was controlled at 1.5 to 2.0. Postoperative enhanced computed tomography revealed good patency of the reconstructed graft 2 months after transplantation (Fig. 3). During 2 years of follow-up, the patient has done well, with good systolic and diastolic function of the transplanted heart and no adverse events.

## Discussion

There are various types of PLSVC. In 92% of patients, the PLSVC connects to the RA via the coronary sinus (CS) and 65% of patients have an absent LBCV [6]. Our patient had a left SVC and atresia of the LBCV, which drained from the left jugular vein and left subclavian vein into the CS.

There are various opinions concerning the best way to deal with PLSVC in heart transplantation, depending on the type of PLSVC. In transplant patients with a right SVC and LBCV of adequate size, simple ligation of the PLSVC is acceptable [4]. If the LBCV is absent, the PLSVC should not be simply ligated because this could cause venostasis repercussions, including facial and left upper arm edema [7]. Therefore, several surgical approaches to reconstruct the PLSVC have been reported, including the use of a prosthetic vascular graft [3]; modification of the recipient’s cardiectomy with preservation of the CS, which is connected to the RA [4]; or harvesting of the donor heart with a long SVC, which is anastomosed to the PLSVC [5].

In our patient, we initially planned to ligate the PLSVC because the right SVC seemed to be larger than the PLSVC. We deemed PLSVC ligation a safe procedure because the innominate vein is sometimes simply ligated and divided to improve access (for example, in aortic arch surgery) without postoperative left upper limb swelling or neurological symptoms [8]. The method of harvesting the donor heart could not be modified in this case because the harvesting procedure preceded the recipient operation. We could not perform modified cardiectomy in the recipient operation because of hard adhesions around the heart, especially on the posterior wall that included the CS, which led to the bridging LVAD implantation while awaiting a donor.

Acute thrombosis and occlusion of the vascular graft is one of the possible postoperative complications and may cause symptoms of acute venostasis. Fortunately, this complication was not observed because we used warfarin to maintain our patient’s international normalized ratio of prothrombin time at 1.5 to 2.0. Postoperative enhanced computed tomography revealed good patency of the reconstructed graft, and the patient had experienced no venostasis symptoms.

Thrombotic occlusion of a prosthetic graft anastomosed to the venous system often occurs, regardless of graft size. However, several studies reported that severe complications, such as pulmonary embolism, were rarely observed [9–11]. Although there is no established anticoagulant therapy after prosthetic graft anastomosis to the venous system, warfarinization for 3 to 6 months after surgery has been proposed in several reports [9–11]. The main purpose of PLSVC reconstruction and warfarinization in our patient was to stabilize the perioperative circulation. We predicted that gradual thrombotic occlusion of the graft might occur despite warfarinization. Long-term gradual occlusion of the graft should not cause problems because adequate collateral circulation will likely develop over time. We think that termination of warfarinization at that time could be justified.

Prosthetic graft infection is another possible adverse event because patients must take immunosuppressive agents after heart transplantation. Although our patient has had no episodes of infection to date, careful follow-up will continue to be necessary.

The advantages of PLSVC reconstruction with a prosthetic vascular graft are that there is no need to change the operative technique on either the donor or recipient side and the procedure is easy to perform. In this case, we did consider anatomical bypass to the right SVC. However, unfortunately, the PLSVC was located behind the left pulmonary artery and in front of the left pulmonary vein. In this situation, we were afraid of graft kinking and/or compression of the pulmonary artery and right ventricle if the reconstructed graft was routed in front of the heart. Therefore, we chose posterior routing as an alternative path. To be sure, the posterior routing risked compression of the pulmonary vein and also had a risk of graft compression by the heart. Therefore, we first anastomosed the ringed graft to the edge of the PLSVC. Then, we checked the graft configuration, including surrounding tissue, and decided to pass the graft posterior to the heart to avoid graft kinking and compression of the pulmonary artery and right ventricle. We thought that anastomosing the graft to the RA free-wall would be easy. Then, we confirmed adequate blood flow through the PLSVC, adjusted the length of the graft posterior to the heart, and performed end-to-side anastomosis. If we had selected anterior graft routing, a longer prosthetic graft would have been needed to avoid heart compression. Another concern is that adhesions around the LV resulting from the prosthetic vascular graft may induce LV diastolic dysfunction. In our patient, follow-up echocardiography 2 years after transplantation showed good systolic and diastolic function of the transplanted heart. However, careful monitoring of cardiac function will be necessary hereafter.

## Conclusions

We successfully performed heart transplantation with PLSVC reconstruction using a prosthetic vascular graft. We were deeply concerned about the risk of complications. However, the patient’s postoperative course has been uneventful 2 years after the surgery, without any venostasis-related symptoms, infections, or diastolic dysfunction. Although careful observation is mandatory, reconstruction of the PLSVC with a prosthetic vascular graft is one option in heart transplantation.

## Data Availability

The authors declare that all data in this article are available within this published article.

## Abbreviations

PLSVC: Persistent left superior vena cava
LV: Left ventricular
LVAD: Left ventricular assist device
LBCV: Left brachiocephalic vein
RA: Right atrium
CS: Coronary sinus
RV: Right ventricle
PA: Pulmonary artery
